# The Impact of COVID-19 Protocols on the Continuity of Care for Patients with Hypertension

**DOI:** 10.3390/ijerph19031735

**Published:** 2022-02-02

**Authors:** Seo Yoon Lee, Sung Youn Chun, Hyeki Park

**Affiliations:** 1 Department of Population Health Nursing Science, College of Nursing, University of Illinois at Chicago, Chicago, IL 60607, USA; slee684@uic.edu; 2 Research and Analysis Team, National Health Insurance Service Ilsan Hospital, Goyang 10444, Korea; cedric6909@nhimc.or.kr; 3 HIRA Research Institute, Health Insurance Review and Assessment Service, Wonju 26465, Korea

**Keywords:** continuity of care, most frequent provider continuity, social distancing, social isolation, primary care, telemedicine

## Abstract

The aim of this study was to investigate the impact of the coronavirus disease 2019 (COVID-19) pandemic on the continuity of care (COC) for patients with hypertension. Additionally, the factor of whether participants were treated via telemedicine was also considered. This study used the National Health Insurance and Medical Aid claims data of the Republic of Korea between 2019 and 2020. Multivariable regression analysis was performed to identify the differences in the number of visits and the most frequent provider continuity (MFPC) of hypertensive patients before and after the appearance of COVID-19 in Korea. Additional analysis was performed with data that excluded cases of patients who received telemedicine services. A total of 5,791,812 hypertensive patients were included in this study. The MFPC decreased by 0.0031 points after the appearance of COVID-19, and it showed the same decrease even when telemedicine cases were excluded. The number of outpatient clinic visit days decreased by 0.2930 days after the appearance of COVID-19. Without the telemedicine cases, the number of outpatient clinic visit days decreased by 0.3330 days after the appearance of COVID-19. Accordingly, the COVID-19 protocols did not affect hypertension patients’ COC but impacted the frequency of their outpatient visits. In other words, with or without telemedicine, the utilization of healthcare was not disrupted, but there was a significant difference in the volume of healthcare use depending on the inclusion of telemedicine cases.

## 1. Introduction

Hypertension is a chronic disease that requires routine management by healthcare providers. The prevalence of hypertension in Korea has increased due to the aging population, Westernized eating habits, and high sodium consumption. Among the population aged over 20 years, the estimated prevalence of hypertension is 29%, and among the patients diagnosed with hypertension, the control rate is only 47% [1]. High blood pressure is the most important modifiable risk factor with the most significant influence on cardiovascular or cerebrovascular disease [2,3]. According to statistics from the National Health Insurance Service (NHIS) in Korea, the estimated medical cost of treating hypertension was KRW 3.83 trillion (i.e., USD 3.4 billion) in 2019. This amount is equivalent to 4% of all medical expenses and 16% of medical expenses for chronic diseases during the same period [1,3]. Thus, controlling the prevalence of hypertension through continuous monitoring and management would reduce the tremendous financial burden caused by the disease and eventually improve patients’ quality of life.

According to Shortell [4], continuity of care (COC) is defined as “the extent to which services are received as part of a coordinated and uninterrupted succession of events consistent with the medical care needs of patients.” It is well recognized that patients receiving continuous care from regular healthcare providers have better health outcomes and higher patient satisfaction at a lower cost [5]. A lower risk of hospital admission has been reported to be associated with higher COC for hypertension patients [6]. Additionally, COC is also associated with a decreased use of emergency room visits and better follow-up rates with appointments [7,8]. This could be because the rapport built with patients allows healthcare providers to have better knowledge about the patients and instantly recognize the changes in their health status. This can eventually prevent the patients from suffering from complications or disease onsets. Thus, COC is an important concept in the control of high blood pressure.

On 11 March 2020, the World Health Organization declared the prevalence of coronavirus disease 2019 (COVID-19) qualified as a pandemic. In response to the pandemic declaration, social distancing and isolation were recommended and implemented to prevent the virus from spreading. Many researchers showed concerns of a negative impact of social isolation on outpatient and primary care [9,10]. Disruption of access to care and preventive measures for patients, as well as delays or postponement of patients’ hospital visits, can have serious implications, which made the implementation of social isolation a significant public health matter to consider. However, telemedicine was identified as an effective method for treating patients in such a situation [11]. The Korean Government also temporarily approved the provision of medical counseling and prescriptions in a non-face-to-face method, i.e., over the telephone. Considering the fact that the unprecedented length of the social isolation period significantly impacts the health outcomes of patients who require continuous care by healthcare providers, this study might be the first to provide information about the influence of a pandemic on the COC of hypertension patients and how telemedicine may reduce the impact of a pandemic in South Korea. Thus, this study aimed to investigate the impact of the COVID-19 pandemic protocols on the COC for hypertension patients. Additionally, patients’ use of telemedicine was considered as part of the investigation.

## 2. Materials and Methods

### 2.1. Source of Data

This study analyzed the use of healthcare by patients with hypertension, using the outpatient claims data of the National Health Insurance (NHI) and Medical Aid beneficiaries reviewed by the Health Insurance Review and Assessment Service (HIRA) in Korea. Since Korea employs a universal health insurance model, all Koreans are mandatorily insured by either NHI or Medical Aid. In order to evaluate the impact of the SARS-CoV-2 outbreak, the cases treated between 20 January 2020, i.e., the first COVID-19 case in Korea, and 31 December 2020 were considered in the study. For comparison, the cases between 20 January and 31 December 2019 were considered. To compare the COC under the same conditions, the data were limited to cases reviewed by March of the following year.

Among those receiving inpatient or outpatient treatment for hypertension (according to the seventh revision of the Korean Standard Classification of Diseases codes, KCD-7: I10, I11, I12, I13) as their major diagnosis or sub-diagnosis, patients who were prescribed antihypertensive drugs twice or more on different days and whose total number of administration days was seven days or more between January and December 2019 were defined as hypertensive patients. Of these, the total number of the study population was 5,791,812, excluding fatalities before 31 December 2020 and those who did not meet the study conditions. This study was exempt from review by the institutional review board of HIRA due to the data used being de-identified data that were made available for public use by HIRA.

### 2.2. Variables

#### 2.2.1. Dependent variables

##### Number of Visits

The frequency of visits to a medical institution is a representative variable that can measure the volume of medical use. It is suitable for measuring changes in medical usage brought on by the COVID-19 protocols due to its consistency compared with medical expenses, which are affected by medical practices. In this study, the number of outpatient visits made due to hypertension as the major diagnosis or sub-diagnosis during each study period was defined as the number of visits.

##### Most Frequent Provider Continuity (MFPC)

In chronic disease management, the continuity of the relationship between the doctor and patient, a type of COC, is known to have a positive effect on the treatment process, outcome, and cost. MFPC is a representative tool that can be used to measure the COC when a patient does not have a designated primary care physician [12]. In this study, the MFPC during each study period was calculated by using the total number of visits to medical institutions due to hypertension as the denominator and the number of visits to the most frequently visited institution as the numerator. The MFPC has a value between 0 and 1, and the closer it is to 1, the higher the COC. If the frequency of visits is too low, there is a possibility that the MFPC calculated will be too high. Therefore, those who visited medical institutions less than twice a year were excluded.

#### 2.2.2. Study Periods

To evaluate the impact of the SARS-CoV-2 outbreak on the patients’ medical use behavior, the period between 20 January 2020, i.e., when the first COVID-19 case was confirmed in Korea, and 31 December 2020 was defined as the period after the introduction of COVID-19 into Korea (the “after period”). The same period in the previous year, with no COVID-19, was defined as the period before the introduction of COVID-19 in Korea (the “before period”).

#### 2.2.3. Independent Variables

The variables with an impact on medical use, such as the patient’s gender, age, region of residence, insurance type, Charlson Comorbidity Index (CCI), diabetes status, duration of hypertension, and type of medical institution most frequently used were adjusted. Any change in the region of residence or insurance type during the study period led to the exclusion of the patient from the study.

Insurance types were classified into National Health Insurance and Medical Aid. National Health Insurance is compulsory public insurance for Korean citizens with a certain level of income or assets, and Medical Aid is a public assistance system for low-income citizens [13]. Therefore, the type of insurance can represent the level of income, which is known to affect one’s health status.

In Korea, there are relatively few restrictions on the use of secondary and tertiary medical institutions for patients with mild conditions; therefore, the proportion of patients with chronic diseases, such as hypertension, who use secondary and tertiary medical institutions, is high [14]. According to a previous study, there was a difference in which medical institutions were mainly used in an epidemic as opposed to a non-epidemic situation [15]. Therefore, we divided the types of medical institutions into clinics, public health centers, general hospitals, and tertiary hospitals based on the type of medical institution mainly used by patients before the SARS-CoV-2 outbreak.

CCI is a representative tool that can measure the severity of patients’ underlying conditions from administrative data, such as health insurance claims data, which lack clinical information [16]. Nineteen of the comorbidities included in the CCI calculation had been included in the inpatient and outpatient claims data during the year before each study period as a major diagnosis or sub-diagnosis (weights of one to six points were given to each morbidity and calculated). A higher score implies a more serious underlying condition. Diabetes mellitus was initially included in the CCI but was excluded because it was measured separately in this study. The presence of diabetes mellitus was defined as KCD-7 E10 to E14 being claimed as a major diagnosis or sub-diagnosis in the inpatient and outpatient claims data during the year before the study period.

### 2.3. Statistical Analyses

In this study, chi-square tests and independent *t*-tests were performed to identify the general characteristics of the study population. Multivariable regression analysis and the independent *t*-test were performed to identify the differences in the number of visits to medical institutions and the MFPC of hypertensive patients before and after the introduction of COVID-19 in Korea. The multivariable regression analysis was adjusted for the patient’s gender, age, region of residence, insurance type, CCI, diabetes status, duration of hypertension, and type of medical institution most frequently used. In consideration of repeated measurements, a generalized estimating equation model was used, and the CORRW type was applied for the covariance structure. In addition, to confirm the effect of telemedicine (contactless treatment in the form of consultations and prescriptions over the phone is used by the Korean Government), which has been temporarily permitted since 24 February 2020 because of the COVID-19 pandemic, additional analysis was performed with data that excluded the telemedicine cases. SAS Enterprise Guide 7.1 was used for all statistical analyses.

## 3. Results

### 3.1. Characteristics of the Study Population

Table 1 shows the general characteristics of the study population. A total of 5,791,812 hypertensive patients, 2,826,779 males and 2,965,033 females, were included. During the before period, those aged 60 to 74 accounted for the highest proportion of the study population at 42.25% (2,446,950 in number). Among the study case population, 1,394,230 patients (24.07%) had diabetes, and those who had had hypertension for more than 10 years (2,978,121 or 51.42%) were among the majority, with a mean CCI of 0.6347. As for the type of insurance, NHI accounted for the majority, with 5,526,514 beneficiaries (95.42%). By region, Gyeonggi Province had the largest number of residents at 1,366,235 (23.59%), followed by Seoul at 985,945 (17.02%) and Busan at 407,245 (7.03%). The most frequently used medical institutions before the appearance of COVID-19 were clinics (4,537,548, 78.34%), and the proportion increased to 80.76% (4,677,502) after the appearance of COVID-19. After the manifestation of the virus, the proportions of tertiary hospitals, general hospitals, and clinics increased by 0.01 percentage points (%p), 0.03%p, and 2.42%p, respectively, and the proportions of hospitals and public health institutions decreased by 0.09%p and 2.37%p, respectively.

Table 2 shows the MFPC before and after the introduction of COVID-19 in South Korea. The MFPC in the before period was 0.9507, and after the appearance of the virus, it decreased by 0.0024 to 0.9483, which was statistically significant. In terms of patient characteristics, the MFPC significantly decreased in most groups after the introduction of COVID-19, but some groups showed an increase. In terms of age, the MFPC decreased in all groups over 60 years old (60 to 74: −0.0033; 75 and more: −0.0057), while it increased in all groups under 60 years old (0 to 20: +0.0066; 30 to 44: +0.0047; 45 to 59: +0.0008), and it was statistically significant. Based on the duration of hypertension, the MFPC increased from 0.9373 points to 0.9467 points in groups that had had hypertension for less than two years after the appearance of COVID 19, and it decreased significantly in groups with a longer period of hypertension. In terms of region, the MFPC increased in Jeju Island by 0.0039 points, Gyeongsangnam-do by 0.0024 points, Jeollabuk-do by 0.0031 points, and Daegu by 0.0014 points. However, it decreased in other areas, such as Sejong, Chungcheongnam-do, Daejeon, and Incheon, with no statistically significant difference. In terms of the most frequently used medical institution, the MFPC increased in tertiary general hospitals (0.0033 points), general hospitals (0.0009 points), and hospitals (0.0047 points), while it decreased in clinics (−0.0024 points) and public health institutions (−0.0507 points).

Table 3 shows the number of visits per patient before and after the appearance of COVID-19. The number of visit days before the appearance of COVID-19 was 8.3347 days, which showed a statistically significant decrease to 8.1363 days thereafter. In terms of patient characteristics, the number of visits significantly decreased in all population groups, except for the population with the onset of hypertension in the two years before the appearance of COVID-19. In particular, there was a greater decrease in the number of visits among women (before: 8.6337, after: 8.4084), patients aged 75 years and over (before: 9.3258, after: 8.9850), patients with diabetes (before: 9.0337, after: 8.7701), patients who had had hypertension for more than 10 years (before: 8.6758, after: 8.4107), Medical Aid beneficiaries (before: 10.3710, after: 10.1173), residents of Sejong-si (before: 8.5040, after: 8.2302), and those who mainly use public health institutions (before: 6.0694, after: 5.6009).

### 3.2. Multivariable Linear Regression Analysis

Table 4 shows the results of the multivariable regression analysis for MFPC and the number of visits per patient. When adjusted for the confounding variables, the MFPC decreased by 0.0031 points after the appearance of COVID-19, which was statistically significant (*p*-value < 0.0001). It also shows the results of the multivariable regression analysis for MFPC when the telemedicine cases are excluded. When the independent variables were adjusted for, the MFPC after the appearance of COVID-19 showed a statistically significant decrease, the same as when the telemedicine cases were included (β: −0.0031, *p*-value < 0.0001). The number of visit days decreased by 0.2930 days after the appearance of COVID-19, which was statistically significant (*p*-value < 0.0001). The result of multivariable regression analysis on the number of visits per patient when telemedicine cases were excluded is also shown. Upon the adjustment of the independent variables, the number of visits per patient after the appearance of COVID-19 decreased by 0.3330 days, which was 0.04 days lower than when the telemedicine cases were included.

## 4. Discussion

The purpose of the study was to identify the impact of social distancing and isolation due to the COVID-19 pandemic on the COC and outpatient visits of hypertension patients. Considering the fact that there are no family doctors in the Korean healthcare system, this study used MFPC as a proxy of COC. To examine the impact, multivariable regression was conducted to compare MFPC with outpatient visits during the pre- and post-pandemic periods. The study found a significant decrease in the MFPC and outpatient visits during the pandemic. Although both the MFPC and the number of outpatient visits decreased with statistical significance, they varied in volume. 

This study found a decrease in the MFPC of hypertensive patients; however, the size of the beta coefficient was not big enough to be considered as a significant decrease. Additionally, regardless of whether the patients received telephone prescriptions, their MFPC was reported to be the same. MFPC represents the pattern of outpatient care usage [12,17]; thus, the study results showed that even though the pandemic impacted and limited patients’ access to outpatient visits, the pattern of outpatient care usage remained the same. Since MFPC represents the pattern of medical care utilization, its results indicated that the medical care utilization patterns of hypertension patients were not impacted, despite the nationwide implementation of COVID-19 protocols. The majority of previous studies reported the positive impact of COC on a patient’s health behaviors and health outcomes; however, a few studies reported different results. COC is, specifically, associated with positive patient and provider satisfaction [18], reduced emergency room use [8], decreased hospital admissions, and better immunization rates [19]. In contrast, COC by a provider is not associated with blood pressure control [7]. However, there was a study that showed that patients with high COC levels (0.67–1.0) were more likely to have better-controlled blood pressure compared with those with medium (0.40–0.66) or low (0–0.39) COC levels [7]. Better controlled blood pressure in hypertension patients leads to better health outcomes. Considering the results of these previous studies and the average MFPC of about 0.95, even with the COVID-19 protocols in effect, the health outcomes of hypertension patients in Korea can be said to have remained almost the same.

The other result shown in the study was the decrease in the number of outpatient visits. Unlike the MFPC results, the number of outpatient visits made by hypertension patients decreased significantly after the COVID-19 pandemic began. For patients who used telemedicine services, the number of outpatient clinic visits still decreased, but not as much as it did for the patients who did not use telemedicine. The number of outpatient clinic visits reflects the volume of medical use and measures the utilization of healthcare services [20,21]. Due to the COVID-19 protocols, a change in the utilization of healthcare services is inevitable, unlike the medical care usage pattern, which remained largely unaffected. However, when the use of telemedicine services is included as part of the frequency of outpatient visits, the data show that the frequency of outpatient visits did not go down by much. Studies regarding the frequency of outpatient visits showed mixed results. A study reported that too many outpatient visits would increase medical expenses and lead to inefficient treatment, as some of the visits would not be necessary [22]. It should be noted, however, that this result was shown when the patients visited multiple doctors for the same condition. Another study reported a shorter return visit interval to be positively associated with blood pressure control [23]. 

Non-face-to-face medical counseling/prescription via telephone (telemedicine is the term used in this study) was temporarily approved by the Korean Government due to a sudden increase in the number of COVID-19 diagnoses nationwide. This increase happened right after a mass infection occurred when an infected person visited a church in the Daegu-Gyeongbuk region. Some studies reported the positive role of telemedicine and the need for it in the era of infectious disease pandemics [24,25], especially in terms of its effectiveness [11,26,27,28]. In this study, when telemedicine cases were taken into consideration, the pattern of healthcare use was shown not to change due to the COVID-19 protocols, and the use of telemedicine also prevented the level of healthcare utilization from decreasing significantly. The fact that hypertension patients’ COC did not decrease, despite a decrease in their healthcare utilization, serves as evidence that telemedicine services can be an effective measure during the COVID-19 pandemic period.

Several limitations of this study should be noted. First, this study used hospitals’ administrative data for medical reimbursement or billing, which precludes the incorporation of the clinical information. In other words, it is hard to confirm any actual changes in patients’ blood pressure. Thus, in the future, it is necessary to develop a patient dataset that links the administrative data and clinical data on clinical variables, such as lab results. Second, since only outpatient medical use was targeted in this study, a patient who was hospitalized during the follow-up period due to cardiovascular disease, a complication of hypertension, may have been classified as not having received any medical care at all. However, in the case of high-blood-pressure patients with such high severity, it is very important to prevent complications through the continuous use of healthcare. Accordingly, analysis of such cases would provide valuable insight, and future studies on hospitalization due to complications of hypertension are needed. Lastly, demographic and socioeconomic factors influence medical use. However, in this study, only basic demographic variables, such as gender and age, and variables concerning clinical characteristics were included in the analysis because the study used administrative data submitted for health insurance claims review. As a result, there is a limitation in that the patients’ knowledge and attitude toward hypertension are not considered at all. Furthermore, among the demographic variables, variables closely related to access to medical institutions, such as the location of residence and income, were not included. To minimize this limitation, in this study, patients’ health insurance status was used as a proxy for their socioeconomic status and income level, and CCI was used and controlled as a proxy for the participant’s condition. In the future, however, it is necessary to develop a patient dataset that links the administrative data and survey data on social and psychological variables.

Despite its limitations, our study had several strengths. First, the study used nationally representative data. Second, to our knowledge, this study was the first to examine the impact of COVID-19 protocols on hypertension patients’ COC in South Korea. Lastly, including whether the participants were treated by using telemedicine in the analysis enriched the interpretation of the data.

## 5. Conclusions

Our results suggested that COVID-19 protocols did not affect hypertension patients’ COC but did have an impact on the frequency of their outpatient visits. Additionally, when telemedicine cases were taken into consideration, the COC was the same with or without telemedicine. However, the patients’ frequency of outpatient visits showed a smaller decrease when the telemedicine cases were included in the analysis. Future studies should examine inpatient cases to evaluate whether the COVID-19 protocols impacted the admission rates of hypertensive patients due to complications. It is hoped that the information in this study will be a useful reference for further studies of chronic disease patients’ COC during infectious disease pandemics and of telemedicine’s impact.

## Figures and Tables

**Table 1:** General characteristics of the population (cases (%); mean ± standard deviation).

	Total		Period				*p*-Value *
			Before (2019)		After (2020)		
**Gender**							
Male	5,653,558	(48.81)	2,826,779	(48.81)	2,826,779	(48.81)	1.0000
Female	5,930,066	(51.19)	2,965,033	(51.19)	2,965,033	(51.19)	
**Age group (years)**							
75 or more	2,674,728	(23.09)	1,280,358	(22.11)	1,394,370	(24.07)	<0.0001
60 to 74	4,974,598	(42.95)	2,446,950	(42.25)	2,527,648	(43.64)	
45 to 59	3,403,191	(29.38)	1,775,890	(30.66)	1,627,301	(28.10)	
30 to 44	508,882	(4.39)	276,419	(4.77)	232,463	(4.01)	
0 to 29	22,225	(0.19)	12,195	(0.21)	10,030	(0.17)	
**Diabetes mellitus**							
Yes	2,890,161	(24.95)	1,394,230	(24.07)	1,495,931	(25.83)	<0.0001
No	8,693,463	(75.05)	4,397,582	(75.93)	4,295,881	(74.17)	
**Hypertension**							
Less than 2 years	678,704	(5.86)	471,291	(8.14)	207,413	(3.58)	<0.0001
2 to 4 years	2,102,954	(18.15)	1,020,221	(17.61)	1,082,733	(18.69)	
5 to 9 years	2,576,125	(22.24)	1,322,179	(22.83)	1,253,946	(21.65)	
10 years or more	6,225,841	(53.75)	2,978,121	(51.42)	3,247,720	(56.07)	
**CCI**	0.64	±0.94	0.63	±0.92	0.65	±0.95	<0.0001
**Insurance type**							
Medical aid	530,857	(4.58)	265,298	(4.58)	265,559	(4.59)	0.7138
NHI	11,052,767	(95.42)	5,526,514	(95.42)	5,526,253	(95.41)	
**Region (province)**							
Sejong-si	47,252	(0.41)	23,625	(0.41)	23,627	(0.41)	1.0000
Jeju-si	143,228	(1.24)	71,614	(1.24)	71,614	(1.24)	
Gyeongsangnam-do	741,163	(6.40)	370,577	(6.40)	370,586	(6.40)	
Gyeongsangbuk-do	699,928	(6.04)	349,961	(6.04)	349,967	(6.04)	
Jeollanam-do	524,119	(4.52)	262,059	(4.52)	262,060	(4.52)	
Jeollabuk-do	499,754	(4.31)	249,875	(4.31)	249,879	(4.31)	
Chungcheongnam-do	522,166	(4.51)	261,081	(4.51)	261,085	(4.51)	
Chungcheongbuk-do	405,470	(3.50)	202,734	(3.50)	202,736	(3.50)	
Gangwon-do	444,728	(3.84)	222,363	(3.84)	222,365	(3.84)	
Gyeonggi-do	2,732,476	(23.59)	1,366,235	(23.59)	1,366,241	(23.59)	
Ulsan-si	226,247	(1.95)	113,124	(1.95)	113,123	(1.95)	
Daejeon-si	313,209	(2.70)	156,606	(2.70)	156,603	(2.70)	
Gwangju-si	279,906	(2.42)	139,953	(2.42)	139,953	(2.42)	
Daegu-si	549,508	(4.74)	274,755	(4.74)	274,753	(4.74)	
Incheon-si	668,122	(5.77)	334,060	(5.77)	334,062	(5.77)	
Busan-si	814,482	(7.03)	407,245	(7.03)	407,237	(7.03)	
Seoul-si	1,971,866	(17.02)	985,945	(17.02)	985,921	(17.02)	
**Institution type**							
Tertiary hospitals	317,701	(2.74)	158,515	(2.74)	159,186	(2.75)	<0.0001
General hospitals	999,470	(8.63)	498,776	(8.61)	500,694	(8.64)	
Hospitals	644,628	(5.56)	324,860	(5.61)	319,768	(5.52)	
Public health centers	406,775	(3.51)	272,113	(4.70)	134,662	(2.33)	
Clinics	9,215,050	(79.55)	4,537,548	(78.34)	4,677,502	(80.76)	
Total	11,583,624	(100.00)	5,791,812	(100.00)	5,791,812	(100.00)	

* The *p*-value is the result of the chi-square test for frequencies or *t*-test for means between the before and after periods.

**Table 2:** MFPC of the general population (mean ± standard deviation).

	Total		Period				*p*-Value *
			Before (2019)		After (2020)		
**Gender**							
Male	0.9502	±0.1221	0.9515	±0.1207	0.9488	±0.1235	<0.0011
Female	0.9488	±0.1218	0.9499	±0.1204	0.9477	±0.1231	<0.0001
**Age group (years)**							
75 or more	0.9427	±0.1274	0.9457	±0.1241	0.9400	±0.1303	<0.0001
60 to 74	0.9516	±0.1189	0.9533	±0.1167	0.9500	±0.1208	<0.0001
45 to 59	0.9530	±0.1196	0.9526	±0.1200	0.9535	±0.1191	<0.0001
30 to 44	0.9409	±0.1344	0.9387	±0.1364	0.9435	±0.1320	<0.0001
0 to 29	0.9313	±0.1454	0.9284	±0.1477	0.9350	±0.1424	0.0007
**Diabetes mellitus**							
Yes	0.9538	±0.1161	0.9553	±0.1142	0.9524	±0.1178	<0.0001
No	0.9480	±0.1238	0.9492	±0.1224	0.9468	±0.1252	<0.0001
**Hypertension**							
Less than 2 years	0.9402	±0.1327	0.9373	±0.1354	0.9467	±0.1262	<0.0001
2 to 4 years	0.9514	±0.1210	0.9517	±0.1205	0.9510	±0.1215	<0.0001
5 to 9 years	0.9511	±0.1206	0.9523	±0.1193	0.9499	±0.1221	<0.0001
10 years or more	0.9492	±0.1215	0.9517	±0.1184	0.9468	±0.1242	<0.0001
**Insurance type**							
Medical aid	0.9479	±0.1215	0.9486	±0.1207	0.9472	±0.1224	<0.0001
NHI	0.9496	±0.1220	0.9508	±0.1205	0.9483	±0.1234	<0.0001
**Region (province)**							
Sejong-si	0.9426	±0.1301	0.9434	±0.1294	0.9418	±0.1308	0.1912
Jeju-si	0.9530	±0.1174	0.9511	±0.1203	0.9550	±0.1144	<0.0001
Gyeongsangnam-do	0.9531	±0.1173	0.9519	±0.1186	0.9543	±0.1159	<0.0001
Gyeongsangbuk-do	0.9505	±0.1198	0.9529	±0.1171	0.9482	±0.1225	<0.0001
Jeollanam-do	0.9449	±0.1246	0.9475	±0.1212	0.9423	±0.1279	<0.0001
Jeollabuk-do	0.9522	±0.1177	0.9506	±0.1198	0.9537	±0.1156	<0.0001
Chungcheongnam-do	0.9459	±0.1248	0.9461	±0.1248	0.9457	±0.1248	0.2047
Chungcheongbuk-do	0.9484	±0.1226	0.9517	±0.1186	0.9451	±0.1263	<0.0001
Gangwon-do	0.9475	±0.1232	0.9521	±0.1174	0.9430	±0.1286	<0.0001
Gyeonggi-do	0.9475	±0.1251	0.9488	±0.1236	0.9462	±0.1267	<0.0001
Ulsan-si	0.9559	±0.1148	0.9597	±0.1091	0.9521	±0.1201	<0.0001
Daejeon-si	0.9522	±0.1183	0.9526	±0.1180	0.9518	±0.1186	0.0690
Gwangju-si	0.9476	±0.1238	0.9497	±0.1214	0.9456	±0.1260	<0.0001
Daegu-si	0.9560	±0.1134	0.9553	±0.1149	0.9567	±0.1119	<0.0001
Incheon-si	0.9521	±0.1190	0.9522	±0.1191	0.9519	±0.1189	0.1824
Busan-si	0.9531	±0.1182	0.9559	±0.1146	0.9504	±0.1216	<0.0001
Seoul-si	0.9474	±0.1248	0.9485	±0.1235	0.9462	±0.1261	<0.0001
**Institution type**							
Tertiary hospitals	0.9287	±0.1532	0.9271	±0.1545	0.9304	±0.1520	<0.0001
General hospitals	0.9457	±0.1328	0.9453	±0.1329	0.9462	±0.1328	0.0004
Hospitals	0.9365	±0.1398	0.9342	±0.1419	0.9388	±0.1375	<0.0001
Public health centers	0.9269	±0.1392	0.9436	±0.1249	0.8930	±0.1592	<0.0001
Clinics	0.9525	±0.1170	0.9537	±0.1155	0.9513	±0.1184	<0.0001
Total	0.9495	±0.1219	0.9507	±0.1205	0.9483	±0.1233	<0.0001

* The *p*-values are the result of the *t*-test for means of MFPC between the before and after periods.

**Table 3:** Number of outpatient clinic visits per patient (mean ± standard deviation).

	Total		Period				*p*-Value *
			Before (2019)		After (2020)		
**Gender**							
Male	7.9359	±4.9085	8.0210	±4.8419	7.8509	±4.9727	<0.0001
Female	8.5211	±5.3288	8.6337	±5.3613	8.4084	±5.2937	<0.0001
**Age group (years)**							
75 or more	9.1481	±6.4048	9.3258	±6.4997	8.9850	±6.3121	<0.0001
60 to 74	8.2590	±4.9318	8.3717	±4.9426	8.1498	±4.9188	<0.0001
45 to 59	7.6344	±4.2311	7.7310	±4.1811	7.5289	±4.2825	<0.0001
30 to 44	7.2854	±4.3218	7.3500	±4.1215	7.2085	±4.5474	<0.0001
0 to 29	6.9444	±4.6984	7.0664	±4.4038	6.7961	±5.0297	<0.0001
**Diabetes mellitus**							
Yes	8.8972	±5.3672	9.0337	±5.3461	8.7701	±5.3836	<0.0001
No	8.0155	±5.0380	8.1130	±5.0307	7.9156	±5.0434	<0.0001
**Hypertension**							
Less than 2 years	7.7525	±4.1013	7.6707	±4.1057	7.9384	±4.0852	<0.0001
2 to 4 years	7.7778	±4.3948	7.9137	±4.4198	7.6497	±4.3673	<0.0001
5 to 9 years	8.0065	±4.8322	8.1278	±4.8228	7.8786	±4.8387	<0.0001
10 years or more	8.5375	±5.5548	8.6758	±5.5768	8.4107	±5.5316	<0.0001
**Insurance type**							
Medical Aid	10.2440	±7.1895	10.3710	±7.2322	10.1173	±7.1443	<0.0001
NHI	8.1390	±4.9963	8.2369	±4.9791	8.0411	±5.0115	<0.0001
**Region (province)**							
Sejong-si	8.3671	±4.7620	8.5040	±4.8872	8.2302	±4.6295	<0.0001
Jeju-si	8.0476	±4.5007	8.1087	±4.4124	7.9865	±4.5866	<0.0001
Gyeongsangnam-do	8.3869	±4.9862	8.4923	±4.9973	8.2815	±4.9729	<0.0001
Gyeongsangbuk-do	8.7201	±4.9404	8.8362	±4.9922	8.6040	±4.8854	<0.0001
Jeollanam-do	9.2460	±7.0298	9.3577	±7.0874	9.1343	±6.9699	<0.0001
Jeollabuk-do	9.3156	±6.6059	9.3957	±6.5387	9.2355	±6.6716	<0.0001
Chungcheongnam-do	8.6725	±5.7471	8.7796	±5.6281	8.5654	±5.8617	<0.0001
Chungcheongbuk-do	8.5476	±5.0932	8.6793	±5.0696	8.4158	±5.1133	<0.0001
Gangwon-do	7.9901	±4.3045	8.1072	±4.2810	7.8730	±4.3248	<0.0001
Gyeonggi-do	7.7192	±4.5631	7.8172	±4.5204	7.6211	±4.6033	<0.0001
Ulsan-si	7.8877	±4.3847	7.9901	±4.4511	7.7854	±4.3148	<0.0001
Daejeon-si	8.7308	±4.9401	8.8511	±5.0355	8.6105	±4.8399	<0.0001
Gwangju-si	8.9130	±5.9559	8.9953	±6.0133	8.8308	±5.8969	<0.0001
Daegu-si	8.8127	±5.2820	8.9259	±5.2352	8.6994	±5.3259	<0.0001
Incheon-si	8.2082	±4.7261	8.3253	±4.7811	8.0910	±4.6675	<0.0001
Busan-si	8.1732	±4.4690	8.2547	±4.4206	8.0918	±4.5155	<0.0001
Seoul-si	7.8048	±5.2366	7.8836	±5.2198	7.7259	±5.2521	<0.0001
**Institution type**							
Tertiary hospitals	4.9222	±3.2682	5.0216	±3.1665	4.8233	±3.3635	<0.0001
General hospitals	5.6255	±3.3779	5.7621	±3.2843	5.4895	±3.4632	<0.0001
Hospitals	7.0832	±4.5357	7.1699	±4.2724	6.9951	±4.7869	<0.0001
Public health centers	5.9143	±3.0225	6.0694	±3.2127	5.6009	±2.5673	<0.0001
Clinics	8.8159	±5.2848	8.9524	±5.3055	8.6834	±5.2613	<0.0001
Total	8.2355	±5.1363	8.3347	±5.1236	8.1363	±5.1471	<0.0001

* The *p*-value is the result of the *t*-test for the means between the Before and After periods.

**Table 4:** Impact of the COVID-19 protocols on MFPC and the number of outpatient clinic visits per patient.

	MFPC				Outpatient Clinic Visits			
	With Telemedicine		Without Telemedicine		With Telemedicine		Without Telemedicine	
	β	*p*-Value	β	*p*-Value	β	*p*-Value	β	*p*-Value
Intercept	0.939	<0.0001	0.9389	<0.0001	8.0054	<0.0001	8.0105	<0.0001
**Period**								
After (2020)	−0.0031	<0.0001	−0.0031	<0.0001	−0.293	<0.0001	−0.333	<0.0001
Before (2019)	Ref.	-	Ref.	-	Ref.	-	Ref.	-
**Gender**								
Male	0.0003	0.0002	0.0003	<0.0001	−0.2548	<0.0001	−0.2527	<0.0001
Female	Ref.	-	Ref.	-	Ref.	-	Ref.	-
**Age group (years)**								
75 or more	0.0071	<0.0001	0.0072	<0.0001	0.9864	<0.0001	0.9921	<0.0001
60 to 74	0.0148	<0.0001	0.0149	<0.0001	0.3353	<0.0001	0.3385	<0.0001
45 to 59	0.016	<0.0001	0.016	<0.0001	−0.1031	0.003	−0.104	0.0029
30 to 44	0.0052	<0.0001	0.0052	<0.0001	−0.2437	<0.0001	−0.2434	<0.0001
0 to 29	Ref.	-	Ref.	-	Ref.	-	Ref.	-
**Diabetes mellitus**								
Yes	0.0067	<0.0001	0.0067	<0.0001	0.5026	<0.0001	0.5012	<0.0001
No	Ref.	-	Ref.	-	Ref.	-	Ref.	-
**Hypertension**								
Less than 2 years	−0.0102	<0.0001	−0.0101	<0.0001	−0.1023	<0.0001	−0.1043	<0.0001
2 to 4 years	0.0007	<0.0001	0.0007	<0.0001	−0.2898	<0.0001	−0.2874	<0.0001
5 to 9 years	0.0005	<0.0001	0.0005	<0.0001	−0.1826	<0.0001	−0.1818	<0.0001
10 years or more	Ref.	-	Ref.	-	Ref.	-	Ref.	-
**CCI**	−0.0039	<0.0001	−0.0039	<0.0001	0.122	<0.0001	0.1226	<0.0001
**Insurance type**								
Medical Aid	0.0004	0.0214	0.0004	0.0258	1.8853	<0.0001	1.8804	<0.0001
NHI	Ref.	-	Ref.	-	Ref.	-	Ref.	-
**Region (province)**								
Sejong-si	−0.0057	<0.0001	−0.0057	<0.0001	0.4773	<0.0001	0.4713	<0.0001
Jeju-si	0.006	<0.0001	0.006	<0.0001	0.3895	<0.0001	0.3941	<0.0001
Gyeongsangnam-do	0.0075	<0.0001	0.0076	<0.0001	0.8056	<0.0001	0.8034	<0.0001
Gyeongsangbuk-do	0.0042	<0.0001	0.0044	<0.0001	1.0135	<0.0001	0.9992	<0.0001
Jeollanam-do	−0.0002	0.3166	−0.0002	0.3906	1.4879	<0.0001	1.4892	<0.0001
Jeollabuk-do	0.0063	<0.0001	0.0063	<0.0001	1.4908	<0.0001	1.4862	<0.0001
Chungcheongnam-do	−0.0009	<0.0001	−0.0009	<0.0001	0.9117	<0.0001	0.9063	<0.0001
Chungcheongbuk-do	0.0011	<0.0001	0.0012	<0.0001	0.7342	<0.0001	0.7355	<0.0001
Gangwon-do	0.0011	<0.0001	0.0012	<0.0001	0.3207	<0.0001	0.3257	<0.0001
Gyeonggi-do	0.0001	0.6459	0.0001	0.6686	0.0425	<0.0001	0.0414	<0.0001
Ulsan-si	0.0084	<0.0001	0.0084	<0.0001	0.333	<0.0001	0.3265	<0.0001
Daejeon-si	0.0037	<0.0001	0.0037	<0.0001	0.8033	<0.0001	0.7987	<0.0001
Gwangju-si	0.0017	<0.0001	0.0017	<0.0001	1.2873	<0.0001	1.2666	<0.0001
Daegu-si	0.0089	<0.0001	0.0088	<0.0001	0.9177	<0.0001	0.8831	<0.0001
Incheon-si	0.0043	<0.0001	0.0043	<0.0001	0.4702	<0.0001	0.4712	<0.0001
Busan-si	0.0072	<0.0001	0.0074	<0.0001	0.5357	<0.0001	0.5343	<0.0001
Seoul-si	Ref.	-	Ref.	-	Ref.	-	Ref.	-
**Institution type**								
Tertiary hospitals	−0.0227	<0.0001	−0.0232	<0.0001	−3.5721	<0.0001	−3.5519	<0.0001
General hospitals	−0.0065	<0.0001	−0.0068	<0.0001	−3.0503	<0.0001	−3.0425	<0.0001
Hospitals	−0.0167	<0.0001	−0.0168	<0.0001	−1.618	<0.0001	−1.6121	<0.0001
Public health centers	−0.01	<0.0001	−0.0103	<0.0001	−2.1993	<0.0001	−2.2147	<0.0001
Clinics	Ref.	-	Ref.	-	Ref.	-	Ref.	-

## Data Availability

Restrictions apply to the availability of these data. The data were obtained from HIRA and are available at https://opendata.hira.or.kr/home.do with the permission of HIRA (accessed on 1 July 2021).
